# Evaluation of Pleth Variability Index in the Lithotomy Position in Geriatric Patients Undergoing Transurethral Resection of the Prostate

**DOI:** 10.3390/diagnostics15151877

**Published:** 2025-07-26

**Authors:** Leyla Kazancıoğlu, Şule Batçık

**Affiliations:** Department of Anesthesiology and Reanimation, Recep Tayyip Erdoğan University Faculty of Medicine, Rize 53020, Türkiye; sule.batcik@erdogan.edu.tr

**Keywords:** geriatrics, pleth variability index, perfusion index, lithotomy position, spinal anesthesia, TUR-P

## Abstract

**Background/Objectives:** The Pleth Variability Index (PVI) is a non-invasive parameter used to guide fluid management by reflecting respiratory-induced variations in the plethysmographic waveform. While PVI’s reliability in various positions has been studied, data on its behavior in geriatric patients undergoing transurethral resection of the prostate (TUR-P) in the lithotomy position remain limited. This study aimed to evaluate the effect of the lithotomy position on PVI in geriatric versus non-geriatric patients under spinal anesthesia. **Methods:** This prospective observational study included 90 patients undergoing elective TUR-P in the lithotomy position under spinal anesthesia. Patients were divided into geriatric (≥65 years, *n* = 48) and non-geriatric (<65 years, *n* = 42) groups. PVI and Perfusion Index (PI) were recorded at baseline, in the supine position, and in the lithotomy position. Fluid and vasopressor requirements, along with hemodynamic parameters, were also analyzed. **Results:** PVI values at the 5th minute in the lithotomy position were significantly higher in the geriatric group compared to the non-geriatric group (*p* = 0.019). No significant differences were observed in PI values or intraoperative hypotension rates between the groups. Neurological comorbidities were more prevalent in the geriatric group (*p* = 0.025). **Conclusions:** PVI appears to be a more sensitive indicator of fluid responsiveness in elderly patients under spinal anesthesia in the lithotomy position. Its age-dependent variability suggests clinical utility in guiding fluid management in geriatric populations, while the stable hypotension rates support the effectiveness of PVI-guided goal-directed therapy.

## 1. Introduction

According to the World Health Organization, people who are 65 years of age or older are classified as geriatric population. Due to advancements in healthcare services and improvements in living conditions, the geriatric population is steadily increasing, and the need for surgical interventions in this age group has become more common. Transurethral resection of the prostate (TUR-P) is an endoscopic surgical procedure considered the gold standard in the treatment of benign prostatic hyperplasia. This procedure is typically per-formed in the lithotomy position, which can exert significant effects on the hemodynamic system [1].

The lithotomy position increases venous return, leading to an elevation in cardiac output, while simultaneously causing a reduction in functional residual capacity due to upward displacement of the diaphragm. Particularly during prolonged procedures, this position may also contribute to venous stasis and an increased risk of thromboembolism [2]. Additionally, during TUR-P, the absorption of irrigation fluid into the systemic circulation may result in significant electrolyte imbalances and cardiovascular alterations [3].

Maintaining hemodynamic stability in geriatric patients is critically important due to the increased prevalence of comorbidities. Currently, fluid management is often per-formed using invasive methods, which may elevate the risk of complications. Therefore, the use of non-invasive and dynamic monitoring techniques has gained prominence. The Pleth Variability Index (PVI) is a non-invasive parameter that estimates fluid responsiveness by assessing respiratory-induced variations in the plethysmographic waveform via pulse oximetry [4,5].

The reliability of PVI in various patient positions has been investigated. A study conducted in the modified prone position reported that PVI had limited success in predicting fluid responsiveness, whereas arterial-based variables, such as stroke volume variation (SVV) and Pulse Pressure Variation (PPV), provided stronger indicators [6]. In the lithotomy position, it has been shown that patient positioning affects the spread and duration of spinal anesthesia, variables that are directly associated with hemodynamic changes [7]. However, in specific surgical procedures such as TUR-P, particularly in the geriatric population, there is a scarcity of studies evaluating the impact of the lithotomy position on PVI. The extent to which this parameter remains reliable in the context of age-related vascular changes and diminished cardiac reserve has not been comprehensively examined in the existing literature. Addressing this gap, our study offers originality by specifically focusing on the geriatric population.

The objective of this study is to assess the effect of the lithotomy position on the Pleth Variability Index (PVI) in geriatric patients undergoing transurethral resection of the prostate (TUR-P).

## 2. Materials and Methods

**Figure 1:**
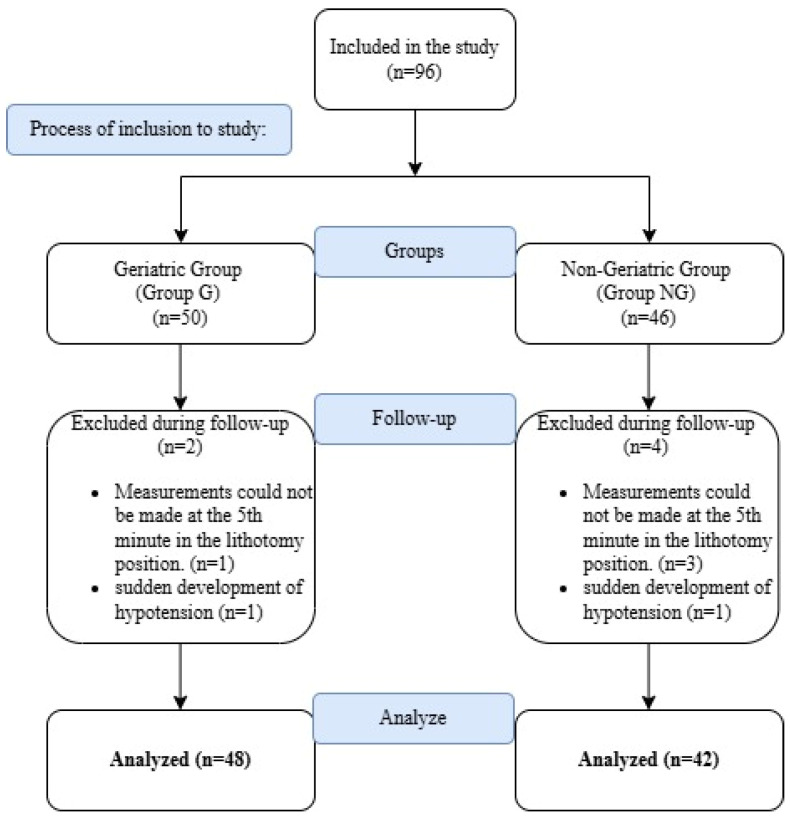
Study flow chart.

Ethical approval for this prospective observational study was obtained from the institutional review board (approval number: 2021/76), prior to its commencement. Prior to inclusion in the study, written informed consent was obtained from each patient. Patients aged between 18 and 84 years, classified as ASA (American Society of Anesthesiologists) physical status I–III, who underwent elective TUR-P surgery under spinal anesthesia in the lithotomy position, were included in the study. Among the 96 patients initially enrolled, four were excluded due to missing measurements at the 5th minute in the lithotomy position, and two were excluded due to the development of sudden hypotension. Data from the remaining 90 patients were analyzed (Figure 1). Subjects were assigned to one of two groups based on study criteria: the geriatric group (Group G, *n* = 48) and the non-geriatric group (Group NG, *n* = 42).

The primary outcome was the comparison of PVI changes over time between geriatric and non-geriatric patients in the lithotomy position. Secondary outcomes included intraoperative fluid requirements, the frequency of vasopressor administration, the trends in PVI and Perfusion Index (PI), and the relationship between these parameters and hemodynamic stability.

Patients were excluded from the study if they were under 18 years of age, had a history of cardiac arrhythmias, presented with a cardiac ejection fraction below 30%, were unable to tolerate a tidal volume greater than 6 mL/kg, had a body mass index (BMI) exceeding 30, were classified as American Society of Anesthesiologists (ASA) physical status IV, exhibited a spinal block level above T8, or had underlying hepatic or renal dysfunction.

Demographic and hemodynamic data collected included ASA physical status scores, comorbidities (diabetes mellitus, chronic obstructive pulmonary disease, coronary artery disease, hypertension, and neurological disorders), and the duration of surgery. Mean arterial pressure (MAP), heart rate (HR), and peripheral oxygen saturation (SpO_2_) were recorded at baseline, at the 5th minute in the supine position, and at the 5th minute in the lithotomy position. Additionally, the occurrence of hypotension (defined as MAP <65 mmHg) was documented.

### 2.1. Spinal Anesthesia Technique

Standard monitoring, including non-invasive arterial blood pressure, electrocardiography and peripheral oxygen saturation (SpO_2_), was applied to all patients preoperatively. Patients were then positioned in the sitting position. After ensuring aseptic conditions, the subarachnoid space was accessed at the L3–L4 or L4–L5 intervertebral level using a 25-gauge Quincke spinal needle via a midline approach. Following the observation of free cerebrospinal fluid (CSF) flow, 15 mg of 0.5% hyperbaric bupivacaine was slowly injected. After the injection, patients were placed in the lithotomy position. The level of the spinal block was assessed using the cold sensation test, and a sensory block up to the T10 dermatome was considered adequate. Supplemental oxygen was administered to all patients at a rate of 2 L/min via nasal cannula.

### 2.2. PVI and PI Measurements

A goal-directed fluid therapy protocol was applied to all patients. As part of this protocol, an intravenous line was established through the left brachial vein, and prior to spinal anesthesia patients received a 500 mL bolus infusion of 0.9% NaCl (isotonic crystalloid solution), followed by maintenance fluid therapy at a rate of 2 mL/kg/h.

For PVI measurements, a pulse oximeter probe from the Radical-7 (Masimo Corp., Irvine, CA, USA) device was placed on the ring finger of the patient’s right hand. Perfusion Index (PI) was also evaluated. PVI and PI values were recorded at three time points: baseline, the 5th minute in the supine position, and the 5th minute in the lithotomy position. A threshold value of 14% was adopted for PVI. This choice is supported by the meta-analysis conducted by Chaves et al. (2024), in which the optimal PVI threshold ranged from 12% to 15%, and several included studies used 14% as the critical cut-off for initiating fluid resuscitation, validating it as an evidence-based parameter [8]. If the PVI value remained at or above 14% for longer than 5 min, a 250 mL bolus infusion of crystalloid fluid was administered intravenously. If the PVI remained at or above 14%, fluid replacement was repeated every 5 min following the same protocol. In cases where mean arterial pressure (MAP) dropped below 65 mmHg, a single intravenous dose of 8 µg norepinephrine was administered; if the heart rate (HR) decreased below 45 beats per minute, 0.5 mg of atropine was administered intravenously.

### 2.3. Sample Size Calculation

The required sample size to compare PVI changes between geriatric and non-geriatric patients in the lithotomy position, was calculated using G*Power 3.1 software (Heinrich Heine University, Düsseldorf, Germany). Based on data from previous similar studies, a moderate effect size (Cohen’s d = 0.5), a 95% confidence level (α = 0.05), and 80% statistical power (1 − β = 0.80) were assumed. According to these parameters, a minimum of 40 patients per group was determined to be necessary. To compensate for potential data loss, a total of 96 patients were enrolled in the study.

### 2.4. Statistical Analysis

All data were statistically analyzed using IBM SPSS Statistics, version 26.0 (Armonk, NY, USA: IBM Corp.). Assessment of the normality of continuous variables was conducted using the Shapiro–Wilk test. Normally distributed data were expressed as mean ± standard deviation (SD). Categorical variables were presented as numbers and percentages (%). For comparisons between groups, the independent samples t-test was used for normally distributed variables. Repeated measures analysis of variance (ANOVA) or the Friedman test was applied to evaluate changes over time. Categorical variables were compared using the chi-square test or Fisher’s exact test as appropriate. Statistical significance was determined in a two-tailed manner, and a *p*-Value of <0.05 was considered statistically significant.

## 3. Results

The mean age of the patients was 73.8 ± 7 years in Group G and 59 ± 4 years in Group NG. There was a statistically remarkable difference in age between the groups (*p* = 0.000, *p* < 0.05). The proportion of ASA I and II patients were significantly higher in Group NG compared to Group G (*p* = 0.000, *p* < 0.05), whereas the proportion of ASA III patients was significantly higher in Group G compared to Group NG (*p* = 0.000, *p* < 0.05). Baseline SpO_2_ levels were also significantly lower in Group G compared to Group NG (*p* = 0.008, *p* < 0.001). No statistically remarkable differences were observed between the groups in the other measured parameters (*p* > 0.05) (Table 1).

The PVI values at the 5th minute in the lithotomy position were significantly higher in Group G compared to Group NG (*p* = 0.019, *p* < 0.05). No notable differences were observed between the two groups in PI values at baseline, at the 5th minute in the supine position, or at the 5th minute in the lithotomy position (*p* > 0.05) (Table 2).

The prevalence of neurological disorders was significantly higher in Group G compared to Group NG (*p* = 0.025, *p* < 0.05) (Table 3).

## 4. Discussion

This study compared hemodynamic responses based on PVI and PI in geriatric and non-geriatric patients placed in the lithotomy position under spinal anesthesia. The findings demonstrate that PVI values were significantly higher at the 5th minute in the lithotomy position in geriatric patients, whereas no statistically notable difference was observed in PI values. These results indicate that positional changes under the effects of spinal anesthesia may exert different impacts on PVI depending on age.

PVI is a non-invasive measure that reflects respiratory-induced variations in dynamic parameters such as peripheral vascular tone, intrathoracic pressure, and volume status. Therefore, it is increasingly being used in anesthesiology for goal-directed fluid management [4,9,10]. In the literature, PVI has been reported to be effective in predicting hypotension in patients undergoing general and spinal anesthesia; however, its predictive value in the geriatric population has been supported by a more limited number of studies [7,10]. Yüksek et al. reported that preoperative PVI values were significantly associated with the development of hypotension in geriatric patients [9]. Similarly, in a study conducted by Zang et al. involving parturient women, PVI was shown to have high sensitivity in predicting hypotension following epidural anesthesia [11]. Kim et al. demonstrated that PVI was useful in predicting fluid responsiveness in the prone position; however, they emphasized that clinical decision-making becomes challenging within the so-called “grey zone” [12]. As demonstrated by Broch et al. (2011), the predictive accuracy of PVI is significantly influenced by factors such as Perfusion Index and physiological variability, highlighting the existence of a ‘grey zone’ where its reliability may be limited [13]. Our study addresses the limitations highlighted in the previous “grey zone” literature by examining geriatric patients undergoing TURP in the lithotomy position under spinal anesthesia—an area where the reliability of PVI has not been thoroughly investigated. By revealing significant differences in PVI values between geriatric and non-geriatric patients, our findings help refine the clinical interpretation of PVI in this specific population. Utilizing a threshold value of 14%, we administered fluid replacement to patients whose PVI values remained above this cut-off, a strategy that may have contributed to maintaining intraoperative hemodynamic stability, particularly in the geriatric group.

The predictive power of PVI becomes more prominent in situations where spinal anesthesia leads to peripheral vasodilation [14]. In the geriatric population, the effectiveness of PVI has been evaluated in patients undergoing surgery for hip fractures while under spinal anesthesia in the surgical position. It was reported that post-spinal hypotension could be associated with either high or low PVI values [15]. This study provides a foundation for further research on the effectiveness of PVI using advanced hemodynamic monitoring in spontaneously breathing patients. Similarly, Sarıhan S et al. [16] assessed the relationship between PVI and other hemodynamic monitoring parameters in patients undergoing lumbar disk herniation surgery under spinal anesthesia. They concluded that in spontaneously breathing patients, PVI could serve as a method capable of quickly and continuously providing insights into blood flow and cardiac output without the need for additional invasive monitoring by establishing reliable threshold values. In geriatric individuals, decreased vascular elasticity and slowed autonomic responses make cardiovascular adaptation during positional changes more challenging [17,18]. This may amplify the effects of vasodilation induced by spinal anesthesia. In our study, the observed increase in PVI values following the lithotomy position in geriatric patients suggests that changes in venous return in this age group may more rapidly lead to fluid deficits, thereby indicating that PVI could have greater clinical significance in the geriatric population.

Although the proportion of ASA III risk group patients was significantly higher in the geriatric group, the intraoperative hypotension rates were found to be similar to those of the non-geriatric group, which is a noteworthy finding. This result highlights the effectiveness of the goal-directed fluid therapy protocol implemented in our study. Previous studies have demonstrated that PVI-based fluid management is effective in maintaining hemodynamic stability [19,20]. Particularly under spinal anesthesia, such protocols may enhance patient safety by reducing the need for invasive monitoring techniques.

Another notable finding in our study was the significantly higher prevalence of neurological disorders in the geriatric group. Neurological diseases are associated with autonomic dysfunction, which can impair blood pressure and heart rate regulation. Conditions such as Parkinson’s disease, dementia, and cerebrovascular diseases may lead to impaired baroreceptor responses, thereby increasing hemodynamic variability following spinal anesthesia [21,22]. This observation suggests that the elevated PVI values observed in the geriatric group may be associated not only with aging itself but also with concomitant neurological conditions.

Maintaining hemodynamic stability in patients undergoing spinal anesthesia is of critical clinical importance. Therefore, the Perfusion Index (PI), as an indicator of peripheral circulation, has attracted attention for its potential to predict early hypotension and perfusion changes. Lal J et al. demonstrated, in their study conducted during caesarean sections, that PI values were significantly associated with hypotension following spinal anesthesia [23]. Similarly, Inamanamelluri R et al. reported that PI could be useful in predicting intraoperative hypotension in patients undergoing caesarean delivery under spinal anesthesia [24]. The study by Jabarulla R et al. also supported the capacity of PI to reflect hemodynamic changes occurring during spinal anesthesia [25]. In our study, the absence of a significant difference in PI values between the groups during the lithotomy position under spinal anesthesia suggests that this parameter may have limited sensitivity to positional changes. This finding indicates that PI alone may not be sufficient for monitoring perfusion changes associated with patient positioning.

The lower baseline SpO_2_ levels observed in the geriatric group may represent an important factor in explaining the increase in PVI during the lithotomy position. Age-related impairments in oxygenation and diminished baroreceptor responsiveness may lead to more pronounced hemodynamic responses to positional changes [26,27,28]. This finding suggests that PVI could serve as a more sensitive and meaningful tool for fluid management in geriatric individuals. It also supports the notion that increased circulatory fragility and decreased oxygen saturation associated with aging may significantly influence PVI values.

Several limitations should be acknowledged in this study. First, being a single-center study with a limited sample size restricts the generalizability of the findings. Additionally, invasive hemodynamic monitoring methods (such as stroke volume variation or cardiac output measurements) were not utilized, and assessments were based solely on non-invasive parameters. This may have limited the ability to fully explain certain physiological changes. Furthermore, the impact of neurological diseases on PVI was not analyzed in detail. Future multicentered, randomized controlled studies with larger sample sizes are needed to validate and expand upon these findings.

## 5. Conclusions

Our study demonstrated that PVI values were significantly higher in geriatric patients compared to non-geriatric patients when placed in the lithotomy position under spinal anesthesia. In contrast, PI values did not differ between the age groups. These findings suggest that PVI is an age-sensitive hemodynamic parameter and should be carefully considered in clinical decision-making processes related to fluid management in geriatric individuals. The absence of a significant difference in intraoperative hypotension rates supports the effectiveness of the goal-directed fluid management protocol. Careful monitoring of PVI threshold values and guiding fluid replacement strategies accordingly may enhance perioperative safety, particularly in elderly patients.

Importantly, our results indicate that incorporating PVI-guided fluid therapy into intraoperative protocols may help maintain hemodynamic stability in the geriatric population. In our study, neurological comorbidities were more prevalent in the geriatric group, which is an expected finding due to age-related disease patterns. Future prospective, outcome-based trials are warranted to evaluate whether PVI performs reliably in elderly patients with neurological conditions, and to determine its potential role in reducing postoperative complications, shortening recovery times, and improving surgical outcomes in this vulnerable population.

## Figures and Tables

**Table 1:** Demographic and Hemodynamic Data.

	Group G (*n* = 48)	Group NG (*n* = 42)	*p* Value
**Preoperative Data**			
Age, years	73.0 ± 7.0	59 ± 4.0	0.000 *
BMI, kg/m^2^	24.5 ± 3.0	25.0 ± 3.5	0.448
ASA Scores, *n*			
I	0	8	0.000 *
II	17	26	0.000 *
III	31	8	0.000 *
**Intraoperative Data**			
Operation duration, min	68 ± 17	68.0 ± 17	0.874
Heart Rate, bpm			
Basal	77 ± 12	79 ± 14	0.431
Supine 5th min	78 ± 16	78 ± 16	0.084
Lithotomy 5th min	75 ± 18	76 ± 16	0.870
MAP, mmHg			
Basal	90 ± 19	98 ± 20	0.054
Supine 5th min	84 ± 22	81 ± 18	0.527
Lithotomy 5th min	88 ± 21	85 ± 17	0.556
SpO_2_, %			
Basal	96 ± 2	97 ± 2	0.008 **
Supine 5th min	97 ± 2	97 ± 2	0.202
Lithotomy 5th min	98 ± 2	98 ± 2	0.416

Group G: Geriatric (>65 years) group, Group NG: Non-Geriatric (<65years) group, BMI: Body mass index, ASA: American Society of Anesthesiologists, bpm: beat per minute, MAP: Mean arterial pressure, SpO_2_: Peripheral oxygen saturation, Values are expressed as mean ± standard deviation. * *p* < 0.05, ** *p* < 0.001.

**Table 2:** PVI and PI measurements.

	Group G (*n* = 48)	Group NG (*n* = 42)	*p* Value
**PVI, %**			
Basal	22 ± 10	21 ± 9	0.342
Supine 5th min	21 ± 8	20 ± 8	0.433
Lithotomy 5th min	19 ± 7	16 ± 7	0.019 *
**PI, %**			
Basal	4 ± 2	4 ± 2	0.902
Supine 5th min	4 ± 2	4 ± 2	0.485
Lithotomy 5th min	4 ± 2	4 ± 2	0.715
Developing Hypotension, *n*	13	15	0.256

Group G: Geriatric (>65 years) group, Group NG: Non-Geriatric (<65 years) group, PVI: Pleth Variability Index, PI: Perfusion Index, Values are expressed as mean ± standard deviation. * *p* < 0.05.

**Table 3:** Comorbidities.

	Group G (*n* = 48)		Group NG (*n* = 42)		*p* Value
	Positive	Negative	Positive	Negative	
DM, *n*	16	32	9	33	0.244
COPD, *n*	12	36	7	35	0.439
CAD, *n*	12	36	6	36	0.292
HT, *n*	21	27	16	26	0.670
ND, *n*	13	35	3	39	0.025 *

DM: Diabetes Mellitus, COPD: Chronic Obstructive Pulmonary Disease, CAD: Coronary Arterial Disease, HT: Hypertension, ND: Neurological Diseases, Group G: Geriatric (>65 years) group, Group NG: Non-Geriatric (<65 years) group, positive: Number of patients with relevant comorbidity, negative: Number of patients without relevant comorbidity, chi-square test, * *p* < 0.05.

## Data Availability

Dataset available on request from the authors.

## Abbreviations

The following abbreviations are used in this manuscript:

ANOVA	Analysis of Variance
ASA	American Society of Anesthesiologists
BMI	Body Mass Index
CSF	Cerebrospinal Fluid
HR	Heart Rate
MAP	Mean Arterial Pressure
PI	Perfusion Index
PPV	Pulse Pressure Variation
PVI	Pleth Variability Index
SVV	Stroke Volume Variation
TUR-P	Transurethral resection of the prostate
